# Testicular Metastasis from Renal Cell Carcinoma: A Systematic Review

**DOI:** 10.3390/jcm12175636

**Published:** 2023-08-29

**Authors:** Anna Pliszka, Sebastian Rajda, Agata Wawrzyniak, Jerzy Walocha, Michał Polguj, Grzegorz Wysiadecki, Edward Clarke, Michał Golberg, Michał Zarzecki, Krzysztof Balawender

**Affiliations:** 1 Department of Normal and Clinical Anatomy, Institute of Medical Sciences, Medical College of Rzeszow University, 35-315 Rzeszow, Poland; annapliszka96@gmail.com (A.P.); sebastianr872@gmail.com (S.R.); 2 Department of Histology and Embryology, Institute of Medical Sciences, Medical College of Rzeszow University, 35-315 Rzeszow, Poland; awawrzyniak@ur.edu.pl; 3 Department of Anatomy, Jagiellonian University Medical College Cracow, 33-332 Kraków, Poland; j.walocha@uj.edu.pl; 4 Youthoria, Youth Research Organization, 33-332 Kraków, Poland; 5 Department of Normal and Clinical Anatomy, Medical University of Lodz, 90-752 Łódź, Poland; michal.polguj@umed.lodz.pl (M.P.); edwardclarke96@icloud.com (E.C.); 6 Department of Histology and Embryology, Medical University of Lodz, 90-752 Łódź, Poland; michal.golberg@stud.umed.lodz.pl; 7 Department of Neonatology and Neonatal Intensive Care, The Children’s Memorial Health Institute, 04-730 Warsaw, Poland; michal.zarzecki96@gmail.com; 8 Clinical Department of Urology and Urological Oncology, Municipal Hospital in Rzeszow, 35-241 Rzeszow, Poland

**Keywords:** renal cell carcinoma, neoplasm metastasis, testicular tumor

## Abstract

Approximately one-third of renal cell carcinoma (RCC) is recognized in its metastatic stage. This systematic review aimed to summarize knowledge on the occurrence and treatment of testicular RCC metastasis. The literature search was performed by two authors independently, with the use of main electronic medical databases (Science Direct, Web of Science, and PubMed) until March 2023 to identify relevant articles that could potentially contribute to this review. Neither language nor publication dates were set as limits. Although we found a total of 51 case reports, only 31 of them contained all the required information. Testicular metastasis in patients with RCC suggests a late stage of the disease. Moreover, it usually does not present typical systemic or specific symptoms except for swelling and enlargement of the affected testis. Knowledge of the possibility of such variants of RCC metastases will allow a clinician to make an appropriate diagnosis and implement adequate treatment without delay, which is crucial in the management of neoplastic disease.

## 1. Introduction

Renal cell carcinoma (RCC) is the sixth most commonly diagnosed neoplasm and compromises 5% of all cancer diagnoses among men [1]. It is often recognized incidentally in diagnostic imaging tests, as it can be asymptomatic for a prolonged time. RCC appears to be the reason why approximately 30% of cases are diagnosed with severe metastatic disease at the time of diagnosis [2].

Approximately 20–50% of RCC patients with localized disease progress to the metastatic stage (mRCC) [3,4,5] in which the most frequent sites of RCC metastasis are the lungs, skeletal system, liver, and brain [6]. Testes are an extremely rare localization for RCC metastasis, and therefore, only a handful of cases have been described in the literature so far, with the first reported in 1946 [7]. Most of them affected the testis ipsilaterally to primary kidney tumor placement and were prevalent more frequently on the left side [8]. They can be found during or before the primary diagnosis of RCC and may initially resemble primary testicular cancer. Their differential diagnosis with metastases is impossible until tumor tissue from the testis is obtained by orchiectomy or biopsy and histopathological examination is performed. However, the list of reports on this subject still needs to be more significant.

According to the authors’ best knowledge, there has been no such precise, systematic review or meta-analysis published that would evaluate mRCC in the testes. Therefore, this article aims to conduct a systematic review of the existing literature to familiarize clinicians with the possibility of this rare location of RCC metastases in the testes. This knowledge will facilitate a more careful evaluation of the patient, a faster appropriate diagnosis, and, consequently, individually adapted treatment.

## 2. Materials and Methods

### 2.1. Search Strategy

**Figure 1:**
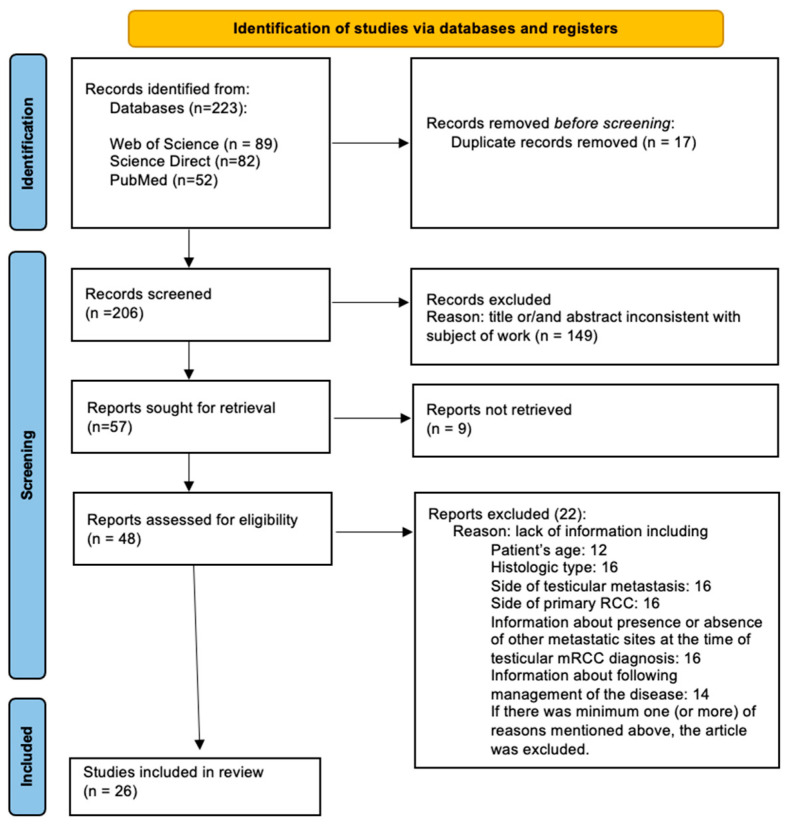
The preferred reporting items for systematic reviews and meta-analyses (PRISMA) flowchart.

The literature search was performed by two authors independently (AP, SR), using the main electronic medical databases (Science Direct, Web of Science, and PubMed) until March 2023 to identify relevant articles that could potentially contribute to this review. The search strategy of this study included the following terms: “testicular renal cell carcinoma metastases”. Non-English articles were translated if appropriate and no date range was introduced. The work was carried out in accordance with the guidelines of the Declaration of Helsinki. Due to the characteristics of this study, it was not necessary to obtain the opinion of the Bioethical Committee. Having obtained the full texts, a reference search was conducted to identify other potentially relevant articles that may have been overlooked in the electronic database. Preferred Reporting Items for Systematic Reviews and Meta-analyses [PRISMA] guidelines were strictly followed in performing this study [9] (Figure 1).

### 2.2. Eligibility Assessment

The research selection and data extraction were split between A.P. and S.R. to allow independent double-checking of articles and data. The inclusion criteria required that studies involve testicular mRCC confirmed by histopathological examination to eliminate any misinterpretation of data due to false diagnosis.

Studies were excluded according to the following criteria:

- Review articles and conference abstracts and studies void of any original data.
- Works that provided incomplete or non-extractable data (such as lack of patient age (12), histological type (16), side of testicular metastasis (16), and side of primary RCC (16); information about the presence or absence of other metastatic sites at the time of diagnosis of testicular mRCC (16), information about follow-up management of the disease (14) or one impossible to extract.

The article was excluded if there was a minimum of one (or more) of the abovementioned reasons.

By contacting the authors of the original studies, any inconsistencies in the included studies were resolved. In each instance where information was not available, all reviewers contributed to the evaluation until consensus was achieved.

### 2.3. Data Extraction

The extraction of data from all studies that met the inclusion criteria for this systematic review was performed individually by two reviewers. The authors of the studies were contacted for clarification or additional information whenever discrepancies in the reported research were found.

### 2.4. Study Endpoints

The analysis is focused on the histological types of renal cell carcinoma with testicular metastasis. The secondary endpoint involved the association between the location of the metastatic tumor in the testes (contralateral vs. ipsilateral) and the location of the primary tumor in the kidney. The tertiary endpoint is concerned with the assessment of the incidence of testicular metastases as the first manifestation of advanced kidney cancer and the presence of other metastases at the stage of diagnosis of metastases in the testes.

### 2.5. Quality Assessment

The Joanna Briggs Institute’s critical evaluation checklist for case reports was used to estimate the quality and reliability of the included studies [10]. Eight domains were evaluated in the analysis: The presence of demographic characteristics of the patient, clear description of the patient’s history presented as a timeline, current clinical condition of the patient, diagnostic tests or assessment methods and results, description of intervention and treatment procedures, identification of adverse events (harms) or unanticipated events, and case takeaway lesson. Each domain was determined in Table 1 with a subjective answer—‘yes’ if the information was present, ‘no’ if it was not provided in the text, ‘unclear’ when the domain information was not fully completed, or ‘not applicable’ if the domain was not related to the article.

### 2.6. Statistical Analysis

#### 2.6.1. Methodology

The significance level of the statistical tests in this analysis was set at α = 0.05. The *p*-value was calculated considering Holm’s correction method for multiple comparisons. The relationships between the two nominal variables were estimated using Fisher’s exact test and Pearson Chi-squared test. A measure of the strength of the relationship, *V*, was calculated in the case of *df* > 1. A comparison of the multiple observed proportions with the expected probabilities was performed using the Chi-squared test for goodness of fit. In the case of two independent groups with nonnormally distributed variables, the Mann–Whitney *U* two-sample rank-sum test was used to compare means. In terms of the effect size measurement, the ${\hat{r}}_{biserial}^{rank}$ was reported. A one-way Kruskal–Wallis ANOVA was used to estimate the differences between the three groups with nonnormally distributed variables. In terms of the effect size measurement, the ${\hat{\epsilon }}_{ordinal}^{2}$ was reported. The central measure of the distribution was reported in the form of the median, and the variability of the distribution in the form of the first and third quartile, *Mdn* (*Q1*–*Q3*). The correlation between the two independent numerical variables ρ (when a variable has at least one outlier) was calculated using the Spearman method. The *p* values were calculated by asymptotic *t approximation.*

#### 2.6.2. Statistical Environment

Analyses were conducted using the R Statistical language (version 4.1.1; R Core Team, 2021) on Windows 10 Pro 64 bit (build 19044), using the packages *sjPlot* (version 2.8.11), *report* (version 0.5.1.3), *rcompanion* (version 2.4.18), *statsExpressions* (version 1.3.5), *ggstatsplot* (version 0.9.3), *psych* (version 2.1.6), and *readxl* (version 1.3.1).

## 3. Results

### 3.1. Study Identification

The process of identifying studies is summarized in Figure 1. The initial search revealed 223 articles that would potentially meet the inclusion criteria. A total of 17 studies were duplicates and were excluded. After the initial screening of abstracts and titles, 206 were considered ineligible: The majority were reviews or reported irrelevant data, while 9 reports were unable to be retrieved. Forty-eight articles were analyzed by full text and, finally, 26 studies were included in this systematic review.

### 3.2. Characteristics of the Included Studies

The characteristics of the included studies are summarized in Table 2. Thirty-one cases were evaluated in this systematic review. The studies included in this research ranged in publication date from January 1984 to March 2023. The geographical distribution of the works was worldwide. The descriptive statistics of the variables on a continuous scale are shown in Table 3. The distributions of the categorical variables are shown in Table 4.

### 3.3. Study Identification

The Joanna Briggs Institute’s critical appraisal checklist for case reports is summarized in Table 1. Nearly half of the articles contained descriptions of the demographic characteristics of the patients. Unclear data were present in approximately 30% overall, while a complete lack of information was in approximately 20%. In most of the articles, case history was explicitly described and depicted as a timeline. Only less than 10% gave unclear or nonchronologically ordered disease details. Similarly, in the largest number of articles, the current clinical condition of the patient was provided on presentation, except in around 15% of them. Data on diagnostic tests or assessment methods with clearly described results and a take-away lesson were present in approximately 90%. Adverse or unanticipated events were not commonly identified and described; they were absent in more than half of the publications and unclear in five. The post-intervention clinical condition was clearly provided in 70% of the articles, while it was unclear in three and lacking such data in five of them.

### 3.4. Age of Testicular Diagnosis of mRCC vs. Testicular Metastasis as the First Clinical Manifestation of RCC

The results of the Mann–Whitney test performed, WMann–Whitney (2) = 133.00, *p* = 0.240, r ^_biserial^rank = 0.27, *n* = 31, showed that there were no significant differences in age at diagnosis of testicular mRCC between patients with testicular metastases as the first clinical manifestation of RCC, Mdn = 65.50 (55.75–68.75) years, *n* = 10, and patients without testicular metastases as the first clinical manifestation of RCC, Mdn = 69.00 (56.00–73.00) years, *n* = 21.

**Figure 2:**
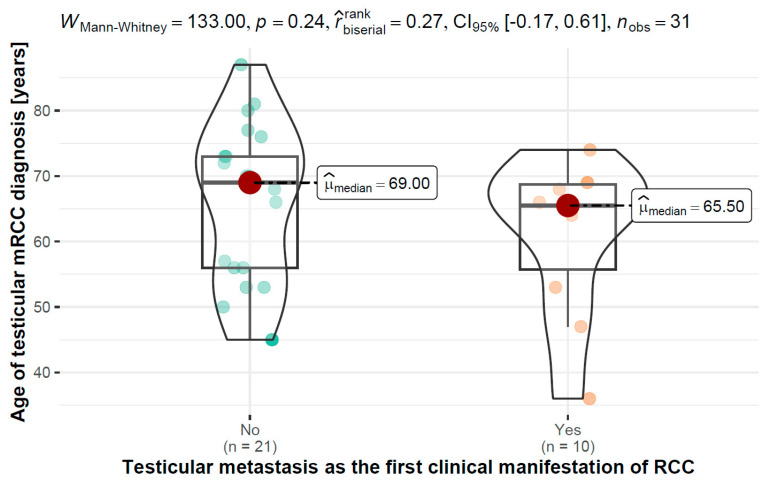
Distribution of age at diagnosis of testicular mRCC between patients with testicular metastases as the first clinical manifestation of RCC and patients without testicular metastases as the first clinical manifestation of RCC.

A graphical visualization of the variable distributions along with the test results can be found in Figure 2.

### 3.5. Age of Testicular mRCC Diagnosis vs.Time Period between Primary Kidney Tumor Diagnosis and Testicular Metastasis

The correlation analysis performed did not show a significant correlation between the age of testicular mRCC diagnosis and the time between the diagnosis of primary kidney tumor and testicular metastasis, ρ = 0.37, *p* = 0.109.

### 3.6. Testicular mRCC Side in Relation to Kidney Primary RCC Side

The results of the Chi-square goodness-of-fit test performed (χ2 = 3.85, df = 1, *p* = 0.049) showed that among the study patients (N = 26), the number of patients with ipsilateral mRCC on the testicular side relative to the primary kidney RCC side (*n* = 18, 69.2%) was significantly higher than the group of patients with contralateral mRCC on the testicular side (*n* = 8, 30.8%).

### 3.7. T Stage of Primary RCC vs. Testicular Metastasis as the First Clinical Manifestation of RCC

The results of the independence test performed, V = 0.56, Fisher’s *p* = 0.620, showed that there was no dependence on frequencies (percentages) between the T stage of the primary RCC site in the kidney and testicular metastasis as the first clinical manifestation of RCC.

### 3.8. T Stage of Primary RCC vs. Time Period between Primary Kidney Tumor Diagnosis and Testicular Metastasis

The results of the Kruskal–Wallis test performed, χ_(Kruskal-Wallis)^2 (2) = 2.44, *p* = 0.300, ϵ ^_ordinal^2 = 0.14, *n* = 18, showed that there were no significant differences in time period between primary kidney tumor diagnosis and testicular metastasis between pT1, Mdn = 36.0 (31.0–69.0) months, *n* = 7, pT2, Mdn = 21.5 (12.0–33.3) months., *n* = 6, and pT3, Mdn = 15.0 (13.0–42.0) months, *n* = 5 stages of primary RCC site in kidney. However, a negative correlation was found between the variables.

**Figure 3:**
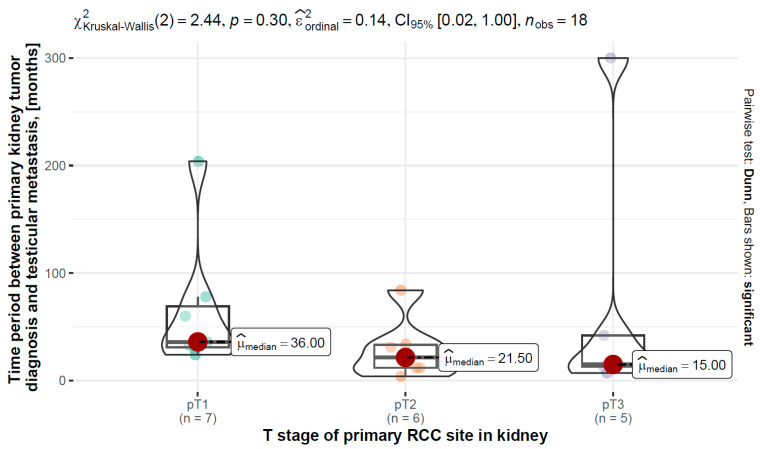
Distribution of time between diagnosis of primary kidney tumor and testicular metastasis in months by T stage of primary RCC site in kidney with the results of the differences between the groups (the pT4 group with fewer than two data points has been omitted).

A graphical visualization of the variable distributions along with the test results can be found in Figure 3.

## 4. Discussion

Patients with RCC develop metastases in 33% of cases, while 25% of them have metastatic disease at the time of diagnosis [35,36].

According to the results of the International mRCC Database Consortium (IMDC), the median age at diagnosis with the mRCC is 60 years, while most of the patients are men (73%). According to our study, the median age of testicular mRCC diagnosis was 68 years. The correlation analysis performed showed no significant relations between the age of diagnosis of testicular mRCC and the time between diagnosis of primary kidney tumor and testicular metastasis. As reported in the previous study, the most common histopathological type is clear cell carcinoma, which occurs in 87% of cases of mRCC [37]. Clear cell RCC also accounted for 87% of all reported cases in our analysis. Basement membrane (BM) and extracellular matrix (ECM) invasion seems to be a key underlying mechanism throughout tumor invasion results in metastasis. Matrix metalloproteinases and heparinase, which are expressed in RCC cells, are known to be responsible for the damage of the major components of the BM and ECM [38]. RCC can affect many sites of the body, but the most common are the lungs (71%), lymph nodes (49%), bone (36%), liver (21%), adrenal (9%), brain (9%), pancreas (5%), pleura (4%), and thyroid (0.6%) [39]. As mentioned above, RCC metastasis to the testis is extremely rare, hence it is not even mentioned in statistics. According to our study, in relation to the primary kidney tumor, more commonly, the ipsilateral testis (69.2%) was affected. Contralateral spreading to the testis was presented in 30.8% of cases. The metastasis was localized in the left gonad in 51.6% of all reported cases, and 3% of the patients had bilateral testicular metastases (Table 2).

RCC is said to spread mainly through blood vessels. The method of expansion of RCC cells into the testes is not certainly clear, but it is believed that they can descend to the gonad through the testicular vein in the case of ipsilateral mRCC or more regionally through the ipsilateral renal capsular veins, then to the Batson’s plexus (the external vertebral venous plexus), contralateral renal capsular veins, and finally spermatic cord vessels and gonad in the case of contralateral mRCC [2].

The median time between primary kidney tumor diagnosis and testicular metastasis was 33.5 months. The relationship between renal tumor stage (T-category based on TNM classification) and time to diagnosis of testicular metastasis was also analyzed. The average time to diagnosis of metastasis for T1, T2, and T3 was 36 months, 21.5 months, and 15 months, respectively.

Prevalent clinical manifestations in patients with RCC (with and without metastases) are irritability with an incidence of 79%, pain and fatigue (71%), worry (71%), and disturbance of sleep (64%), while patients with mRCC more frequently report fatigue (82%), weakness and worry (65%), shortness of breath, and irritability (53%) [38]. Symptoms directly caused by testicular metastasis of RCC are non-characteristic and nonspecific, manifesting as a palpable rigid mass in the scrotum.

Usually, such RCC metastatic manifestation is initially diagnosed as primary independent cancer of the testis, and suspicion of RCC is made on the staging level based on a CT scan, which reveals primary renal tumors. After testicular mRCC resection, the diagnosis is confirmed by its histopathological examination. The disease ought to be differentiated from gonadal germ cell tumor, which is generally the most common type of testis cancer, and testicular lymphoma occurring most frequently among men aged over 70 years [40]. The standard management of testicular tumors is orchiectomy, which provides material for histopathological examination [41]. A CT scan, performed before surgery, enables one to identify the primary location of the disease and other possible sites of metastases. Cytoreductive nephrectomy (CN) is palliative for the majority of patients with mRCC and systemic treatment is required. According to the outcomes of the CARMENA (NCT00930033) and SURTIME (NCT01099423) randomized clinical trials in mRCC patients with intermediate and poor prognosis IMDCs, the primary tumor needs to be treated with immunotherapy-based combination therapy ahead of time. Subsequent cytoreductive nephrectomy may be performed in patients with a clinical response to systemic therapy. In patients ineligible for surgery, mRCC needs to undergo systemic treatment with vascular endothelial growth factor (VEGF) receptor inhibitors, tyrosine kinase inhibitors, and immunotherapies. Cytoreductive nephrectomy with complete resection of a single metastasis or three oligometastases may improve survival and delay systemic therapy, therefore, CN is preferred in patients with oligometastases when complete local treatment of the metastases can be achieved [31,42,43].

Among patients with nonresectable metastatic disease or disqualified for surgery due to poor general condition, embolization can control symptoms, including visible hematuria or flank pain [42].

### Study Limitations

Owing to the casuistic nature of the problem, our systematic review is focused on the case reports or case series evaluation. Moreover, because of the large-scale studies shortage in literature, we were not able to perform a meta-analysis.

## 5. Conclusions

Our review outlines the possibility of the rare metastatic variant appearance of RCC in clinical practice. Generally, such disease is highly probable to manifest in a patient who approaches a urological clinic without systemic or specific symptoms with a suspicion of a primary testicular tumor, confirmed on ultrasound. Standard management is typically based on orchiectomy and postoperative histopathological examination, which reveals the primary severe disease (mRCC). Perceiving even the subtlest cancer symptoms enables one to promptly suspect, recognize, and prevent the tumor from spreading. Symptoms of RCC often appear in advanced metastatic disease, which makes it impossible for early disease identification and proper treatment implementation. We want to emphasize the importance of knowledge of rare manifestations of RCC metastases, such as testicular mRCC, so that a severe cancer process in the patient’s body, especially when the primary disease is clinically silent, is quickly and adequately recognized. In addition, it will also contribute to faster implementation of surgical and systemic treatments.

## Figures and Tables

**Table 1:** The risk of bias analysis based on the Joanna Briggs Institute’s critical appraisal tool.

Case No.	References	Were Patients’ Demographic Characteristics Clearly Described?	Was the Patient’s History Clearly Described and Presented as a Timeline?	Was the Current Clinical Condition of the Patient on Presentation Clearly Described?	Were Diagnostic Tests or Assessment Methods and the Results Clearly Described?	Was the Intervention(s) or Treatment Procedure(s) Clearly Described?	Was the Post-Intervention Clinical Condition Clearly Described?	Were Adverse Events (Harms) or Unanticipated Events Identified and Described?	Does the Case Report Provide Takeaway Lessons?
1.	Bandler and Roen, 1946 [7]	Yes	Yes	Yes	Yes	Yes	No	Yes	Yes
2.	Haupt et al., 1984 [11]	Yes	Yes	Yes	Yes	Yes	Yes	Yes	Yes
3.	Dieckmann, Due and Loy, 1988 [12]	No	No	Yes	Unclear	Yes	Yes	Yes	Yes
4.	Daniels and Schaefer, 1991 [13]	Yes	Yes	Yes	Yes	Yes	Yes	No	Yes
5.	Steiner et al., 1999 [14]	No	Yes	Yes	Yes	Yes	Yes	No	Yes
6.	Camerini et al., 2007 [15]	Yes	Yes	Yes	Yes	Yes	Yes	Yes	Yes
7.	Nemoto, Shimizu, and Kimura, 2007 [16]	Yes	Yes	Yes	Yes	Yes	Yes	Yes	Unclear
8.	Schmorl, Ostertag and Conrad, 2008 [17]	Unclear	Unclear	No	Yes	Yes	Yes	No	Yes
9.	Llarena et al., 2008 [18]	Unclear	Yes	Yes	Yes	Yes	Yes	Unclear	Yes
10.	Wu et al., 2010 [19]	Unclear	Yes	Yes	Yes	Yes	Yes	No	Yes
11.	Moriyama et al., 2014 [20]	Unclear	Yes	Unclear	Yes	Yes	Unclear	No	Yes
12.	Dell’Atti, 2016 [21]	Yes	Yes	Yes	Yes	Yes	Yes	No	Yes
13.	Kongnyuy et al., 2016 [22]	Yes	Yes	Yes	Yes	Yes	Unclear	No	Yes
14.	Libert et al., 2016 [23]	No	Yes	Yes	Yes	Yes	No	No	Yes
15.	Libert et al., 2016 [23]	No	Yes	Yes	Yes	Yes	No	No	Yes
16.	Pichler et al., 2017 [24]	Yes	Yes	Yes	Yes	Yes	Yes	No	Yes
17.	Rouvinov et al., 2017 [25]	Yes	Yes	Yes	Yes	Yes	Unclear	No	Yes
18.	Huang et al., 2018 [26]	No	Yes	Yes	Yes	Yes	Yes	No	Yes
19.	Kolukcu et al., 2019 [27]	Yes	Yes	Yes	Yes	Yes	Yes	No	Yes
20.	De Gobbi et al., 2019 [28]	Unclear	Yes	Yes	Yes	Yes	Yes	Yes	Yes
21.	Gang Wang et al., 2020 [8]	Yes	Yes	Unclear	Yes	Yes	Yes	No	Yes
22.	Gang Wang et al., 2020 [8]	No	Yes	No	No	Yes	No	No	Yes
23.	Gang Wang et al., 2020 [8]	No	Yes	No	No	Unclear	Yes	No	Yes
24.	Gang Wang et al., 2020 [8]	No	No	No	No	Unclear	No	No	Yes
25.	Gang Wang et al., 2020 [8]	No	Yes	No	Unclear	Yes	Yes	Unclear	Yes
26.	De Jonge, Pahernik and Pandey, 2020 [29]	Unclear	Yes	Yes	Yes	Yes	Yes	Yes	Yes
27.	Safriadi, Noegroho and Partogu, 2020 [30]	No	Yes	Yes	Yes	Yes	Yes	No	No
28.	Turco et al., 2021 [31]	Unclear	Yes	Yes	Yes	Yes	Yes	Unclear	Yes
29.	Moradi et al, 2022 [32]	Yes	Yes	Yes	Yes	Yes	No	No	No
30.	Balawender et al., 2023 [33]	Yes	Yes	Yes	Yes	Yes	Yes	Unclear	Yes
31.	Thomson et al., 2023 [34]	No	Yes	Yes	Yes	Yes	Yes	Unclear	Yes

**Table 2:** Characteristics of the cases included in the database in chronological order. mRCC—metastatic renal cell carcinoma; N/A—not available; p—histopathological, tki—tyrosine kinase inhibitor.

Case No.	References	Age of Testicular mRCC Diagnosis [Years]	Histopathological Type	“T” Stage of Primary RCC Site in Kidney	Grading in the Fuhrman Scale	Side	Testicular mRCC Side in Relation to Kidney Primary RCC Side	Other Metastatic Sites Present at the Time of Testicular mRCC Diagnosis	Testicular Metastasis as the First Clinical Manifestation of RCC	Time Period between Primary Kidney Tumor Diagnosis and Testicular Metastasis [months]	Primary Kidney Tumor Treatment	Metastasis Treatment
1.	Bandler and Roen, 1946 [7]	47	unclassified	N/A	N/A	right	ipsilateral	no other metastatic sites present at the time of testicular mRCC diagnosis	Yes	N/A	radical right nephrectomy	right orchiectomy
2.	Haupt et al., 1984 [11]	36	unclassified	N/A	N/A	left	ipsilateral	inguinal lymph nodes	Yes	N/A	chemotherapy (Adriamycin, 5-fluorouracil, vincristine, mitomycin C) with radiotherapy	left orchiectomy
3.	Dieckmann, Due and Loy, 1988 [12]	73	clear cell	pT3a	N/A	left	ipsilateral	lungs	No	13	tumor vessels embolization as palliative treatment	left orchiectomy
4.	Daniels and Schaefer, 1991 [13]	87	clear cell	pT3	N/A	right	contralateral	paraaortic, paravertebral lymph nodes	No	15	left radical nephrectomy	right orchiectomy
5.	Steiner et al., 1999 [14]	66	clear cell	pT1b	N/A	left	contralateral	no other metastatic sites present at the time of testicular mRCC diagnosis	Yes	N/A	right radical nephrectomy	left orchiectomy
6.	Camerini et al., 2007 [15]	45	clear cell	pT2	G2	right	ipsilateral	bones, lungs, pleura	No	4	right radical nephrectomy	right orchiectomy
7.	Nemoto, Shimizu, and Kimura, 2007 [16]	56	clear cell	pT1b	G1	left	ipsilateral	no other metastatic sites present at the time of testicular mRCC diagnosis	No	36	left nephron-sparing surgery followed by left radical nephrectomy	left orchiectomy
8.	Schmorl, Ostertag and Conrad, 2008 [17]	66	clear cell	pT3a	G2	right	ipsilateral	bones	No	42	radical right nephrectomy	immunochemotheraphy (Interferone, Interleukine-2, 5-Fluorouracil)
9.	Llarena et al., 2008 [18]	57	clear cell	pT2	G2	right	ipsilateral	bones	No	12	right radical nephrectomy	immunochemotheraphy (IL-2 inhalations changed for Sorafenib), right orchiectomy
10.	Wu, Hai-Yang et al., 2010 [19]	70	chromophobe renal cell	N/A	N/A	left	contralateral	adrenal glands	No	72	right radical nephrectomy	bilateral adrenalectomy, left orchiectomy
11.	Moriyama et al., 2014 [20]	65	clear cell	pT1b	N/A	bilateral	N/A	pancreas, adrenal glands	Yes	N/A	left nephron-sparing surgery, followed by left radical nephrectomy	bilateral orchiectomy
12.	Dell’Atti, 2016 [21]	69	clear cell	pT1b	N/A	left	ipsilateral	no other metastatic sites present at the time of testicular mRCC diagnosis	No	24	left radical nephrectomy	left orchiectomy
13.	Kongnyuy et al., 2016 [22]	68	clear cell	pT3a	G4	left	contralateral	lungs	Yes	N/A	right radical nephrectomy followed by sunatinib, changed for atixitinib	left orchiectomy
14.	Libert et al., 2016 [23]	69	clear cell	pT1b	N/A	right	ipsilateral	no other metastatic sites present at the time of testicular mRCC diagnosis	Yes	N/A	right radical nephrectomy	right orchiectomy
15.	Libert et al., 2016 [23]	77	clear cell	N/A	N/A	left	ipsilateral	lungs	No	60	left nephron-sparing surgery	left orchiectomy
16.	Pichler et al., 2017 [24]	53	clear cell	pT3b	G3	left	ipsilateral	lungs, liver	Yes (paratesticular metastases with spermatic cord infiltration)	N/A	radical nephrectomy with cavotomy and thrombectomy	chemotherapy (Sunitinib), left orchiectomy
17.	Rouvinov et al., 2017 [25]	72	clear cell	pT1b	N/A	right	ipsilateral	no other metastatic sites present at the time of testicular mRCC diagnosis	No	78	right nephron-sparing surgery	right orchiectomy
18.	Huang et al., 2018 [26]	64	clear cell	pT3a	G2	right	ipsilateral	no other metastatic sites present at the time of testicular mRCC diagnosis	Yes	N/A	right radical nephrectomy	right orchiectomy
19.	Kolucu et al., 2019 [27]	56	clear cell	pT2a	G2	left	contralateral	no other metastatic sites present at the time of testicular mRCC diagnosis	No	12	right radical nephrectomy	chemotherapy (Sunitinib), right orchiectomy
20.	De Gobbi et al., 2019 [28]	53	clear cell	pT3a	G3	left	ipsilateral	lungs	No	7	left radical nephrectomy with thrombectomy	chemotherapy (Sunitinib, changed for programmed cell death protein 1 inhibitors: anti-PD-1 + Nivolumab), left orchiectomy
21.	Gang Wang et al., 2020 [8]	53	clear cell	pT1	G1	bilateral	N/A	no other metastatic sites present at the time of testicular mRCC diagnosis	No	33	tumor vessels embolization followed by left nephron sparing surgery	bilateral orchiectomy
22.	Gang Wang et al., 2020 [8]	81	clear cell	pT2a	G3	left	ipsilateral	bones	No	34	left radical nephrectomy	left orchiectomy
23.	Gang Wang et al., 2020 [8]	45	clear cell	pT2b	G4	right	contralateral	bones	No	31	left radical nephrectomy	right orchiectomy
24.	Gang Wang et al., 2020 [8]	76	clear cell	pT1	G1	right	contralateral	no other metastatic sites present at the time of testicular mRCC diagnosis	No	29	left nephron-sparing surgery followed by left radical nephrectomy	right orchiectomy
25.	Gang Wang et al., 2020 [8]	68	clear cell	pT2b	N/A	left	ipsilateral	bladder, stomach	No	84	left radical nephrectomy	left orchiectiomy, gastric polyp resection, TURBT
26.	De Jonge, Pahernik and Pandey, 2020 [29]	70	clear cell	pT3a	N/A	right	N/A	lungs, thyroid, brain	No	300	right nephrectomy, left nephron sparing surgery	chemotherapy (Interferon/Interleukin, Sunitinib, Nivolumab, experimental arm of the phase III GOLD study: sorafenib vs. Dovitinib), pulmonary metastasectomy, stereotactic radiation, right orchiectomy
27.	Safriadi, Noegroho and Partogu, 2020 [30]	69	papillary	pT2b	N/A	left	ipsilateral	penis, liver	Yes	N/A	left nephrectomy	chemotherapy (Lenvatinib), left orchiectomy, penectomy
28.	Turco et al., 2021 [31]	73	clear cell	pT1a	G1	right	ipsilateral	lungs, liver, pancreas	No	204	right nephron-sparing surgery	chemotherapy (Sunitinib changed for Cabozantinib, then for Nivolumab), left orchiectomy
29.	Moradi et al, 2022 [32]	50	clear cell	pT1b	G3	bilateral	N/A	no other metastatic sites present at the time of testicular mRCC diagnosis	No	No information provided	left nephrectomy	chemotherapy (Sunitinib), bilateral orchiectomy
30.	Balawender et al., 2023 [33]	74	clear cell	pT4	G3	left	N/A	no other metastatic sites present at the time of testicular mRCC diagnosis	Yes	N/A	N/A (qualified to tki therapy but died before treatment)	Left orchiectomy
31.	Thomson et al., 2023 [34]	80	clear cell	pT1a	G2	left	contralateral	no other metastatic sites present at the time of testicular mRCC diagnosis	No	60	right nephrectomy	Left orchiectomy

**Table 3:** The descriptive statistics of the variables. mRCC—metastatic Renal Cell Carcinoma.

*Parameter*	*N*	*Mdn (Q1–Q3)*
Age of diagnosis of testicular mRCC, [years]	31	68.0 (54.5–72.5)
Time period between primary kidney tumor diagnosis and testicular metastasis [months]	20	33.5 (14.5–63.0)

**Table 4:** The distributions of the categorical variables. mRCC—metastatic Renal Cell Carcinoma.

	*N*	*Frequency (%)*
Histopathological type:		
- clear cell; - chromophobe renal cell; - papillary; - unclassified;	31	27 (87.2) 1 (3.2) 1 (3.2) 2 (6.4)
“T” stage of the primary RCC site in the kidney:		
- pT1; - pT1a; - pT1b; - pT2; - pT2a; - pT2b; - pT3; - pT3a - pT3b; - pT4.	27	2 (7.4) 2 (7.4) 7 (25.9) 2 (7.4) 2 (7.4) 3 (11.1) 1 (3.7) 6 (22.3) 1 (3.7) 1 (3.7)
Grading in the Fuhrman scale	17	
- G1; - G2; - G3; - G4		4 (23.5) 6 (35.3) 5 (29.4) 2 (11.8)
Side:		
- bilateral; - left; - right.	31	3 (9.7) 16 (51.6) 12 (38.7)
Testicular mRCC side in relation to kidney primary RCC side:		
- contralateral; - ipsilateral;	26	8 (30.8) 18 (69.2)
Other metastatic sites present at the time of testicular mRCC diagnosis:		
- no other metastatic sites present at the time of testicular mRCC diagnosis; - bones; - lungs; - other.	31	13 (41.9) 5 (16.1) 8 (25.7) 10 (32.3)
Testicular metastasis as the first clinical manifestation of RCC:	31	
- no; - yes		21 (67.7) 10 (32.3)
Primary kidney tumor treatment:		
- unilateral radical nephrectomy; - chemotherapy; - left (right) nephron-sparing surgery; - tumor vessels embolization; - other	31	24 (77.4) 2 (6.5) 8 (25.8) 2 (6.5) 4 (12.9)
Metastasis treatment:		
- unilateral orchiectomy; - bilateral orchiectomy; - chemotherapy; - other	31	27 (87.1) 3 (9.7) 8 (25.8) 4 (12.9)

## Data Availability

All data are contained in the manuscript.

## References

[1] Capitanio U., Bensalah K., Bex A., Boorjian S.A., Bray F., Coleman J., Gore J.L., Sun M., Wood C., Russo P. (2019). Epidemiology of Renal Cell Carcinoma. Eur. Urol..

[2] Marzouk K., Alyami F., Merrimen J., Bagnell S. (2014). Metastatic renal cell carcinoma to the testis: A case report and review of the literature. Can. Urol. Assoc. J..

[3] Mori K., Mostafaei H., Miura N., Karakiewicz P.I., Luzzago S., Schmidinger M., Bruchbacher A., Pradere B., Egawa S., Shariat S.F. (2021). Systemic therapy for metastatic renal cell carcinoma in the first-line setting: A systematic review and network meta-analysis. Cancer Immunol. Immunother..

[4] Gill D.M., Hahn A.W., Hale P., Maughan B.L. (2018). Overview of Current and Future First-Line Systemic Therapy for Metastatic Clear Cell Renal Cell Carcinoma. Curr. Treat. Options Oncol..

[5] Posadas E.M., Limvorasak S., Figlin R.A. (2017). Targeted therapies for renal cell carcinoma. Nat. Rev. Nephrol..

[6] Sivaramakrishna B., Gupta N.P., Wadhwa P., Hemal A.K., Dogra P.N., Seth A., Aron M., Kumar R. (2005). Pattern of metastases in renal cell carcinoma: A single institution study. Indian J. Cancer.

[7] Bandler C.G., Roen P.R. (1946). Solitary Testicular Metastasis Simulating Primary Tumor and Antedating Clinical Hypernephroma of the Kidney: Report of a Case. J. Urol..

[8] Wang G., Zhou C., Villamil C.F., So A., Yuan R., English J.C., Jones E.C. (2020). Metastatic Renal Cell Carcinoma to the Testis: A Clinicopathologic Analysis of Five Cases. Case Rep. Pathol..

[9] Page M.J., Moher D., McKenzie J.E. (2022). Introduction to preferred reporting items for systematic reviews and meta-analyses 2020 and implications for research synthesis methodologists. Res. Synth. Methods.

[10] Moola S., Munn Z., Tufanaru C., Aromataris E., Sears K., Sfetic R., Currie M., Lisy K., Qureshi R., Mattis P. (2019). Chapter 7: Systematic reviews of etiology and risk. JBI Manual for Evidence Synthesis.

[11] Haupt H.M., Mann R.B., Trump D.L., Abeloff M.D. (1984). Metastatic carcinoma involving the testis. Clinical and pathologic distinction from primary testicular neoplasms. Cancer.

[12] Dieckmann K.-P., Düe W., Loy V. (1988). Intrascrotal Metastasis of Renal Cell Carcinoma. Eur. Urol..

[13] Daniels G.F., Schaeffer A.J. (1991). Renal cell carcinoma involving penis and testis: Unusual initial presentations of metastatic disease. Urology.

[14] Steiner D.H.G. (1999). Simultaneous Contralateral Testicular Metastasis from a Renal Clear Cell Carcinoma. Scand. J. Urol. Nephrol..

[15] Camerini A., Tartarelli G., Martini L., Donati S., Puccinelli P., Amoroso D. (2007). Ipsilateral right testicular metastasis from renal cell carcinoma in a responder patient to interleukine-2 treatment. Int. J. Urol..

[16] Nemoto K., Shimizu H., Kimura G. (2007). Testicular metastasis and local recurrence of renal cell carcinoma after nephron-sparing surgery in von Hippel-Lindau disease. Hinyokika Kiyo. Acta Urol. Jpn..

[17] Schmorl P., Ostertag H., Conrad S. (2008). Intratestikuläre Metastasierung eines Nierenzellkarzinoms [Intratesticular metastasis of renal cancer]. Die Urol..

[18] Llarena Ibarguren R., García-Olaverri Rodríguez J., Azurmendi Arin I., Olano Grasa I., Pertusa Peña C. (2008). Metástasis testicular metacrónica secundaria a adenocarcinoma renal de células claras [Metachronic testicular metastasis secondary to clear cell renal adenocarcinoma]. Arch. Esp. Urol..

[19] Wu H.-Y., Xu L.-W., Zhang Y.-Y., Yu Y.-L., Li X.-D., Li G.-H. (2010). Metachronous contralateral testicular and bilateral adrenal metastasis of chromophobe renal cell carcinoma: A case report and review of the literature. J. Zhejiang Univ. B.

[20] Moriyama S., Takeshita H., Adachi A., Arai Y., Higuchi S., Tokairin T., Chiba K., Nakagawa K., Noro A. (2014). Simultaneous bilateral testicular metastases from renal clear cell carcinoma: A case report and review of the literature. Oncol. Lett..

[21] Dell’Atti L. (2016). Unusual Ultrasound Presentation of Testicular Metastasis from Renal Clear Cell Carcinoma. Rare Tumors.

[22] Kongnyuy M., Lawindy S., Martinez D., Parker J., Hall M. (2016). A Rare Case of the Simultaneous, Multifocal, Metastatic Renal Cell Carcinoma to the Ipsilateral Left Testes, Bladder, and Stomach. Case Rep. Urol..

[23] Libert F., Cabri-Wiltzer M., Dardenne E., Draguet A.-P., Puttemans T. (2016). Ultrasonographic Pattern of Testicular Metastasis of Clear Cell Renal Cell Carcinoma with Pathological Correlation. J. Belg. Soc. Radiol..

[24] Pichler R., Tulchiner G., Aigner F., Horninger W., Heidegger I. (2017). Paratesticular Metastasis of a Clear-Cell Renal-Cell Carcinoma With Renal Vein Thrombus Mimicking Primary Testicular Cancer. Clin. Genitourin. Cancer.

[25] Rouvinov K., Neulander E.Z., Kan E., Asali M., Ariad S., Mermershtain W. (2017). Testicular Metastasis from Renal Cell Carcinoma: A Case Report and Review of the Literature. Case Rep. Oncol..

[26] Huang H., Ling W., Qiu T., Luo Y. (2018). Ultrasonographic features of testicular metastasis from renal clear cell carcinoma that mimics a seminoma: A case report. Medicine.

[27] Kolukcu E., Kilic S., Parlaktas B.S., Deresoy F.A., Atilgan D., Gumusay O., Uluocak N. (2019). Contralateral Testicular Metastasis of Renal Cell Carcinoma: A Case Report. Eurasian J. Med..

[28] De Gobbi A., Mangano M.S., Cova G., Lamon C., Maccatrozzo L. (2019). Testicular metastasis from renal cell carcinoma after nephrectomy and on tyrosine kinase inhibitors therapy: Case report and review. Urol. J..

[29] De Jonge L., Pahernik S., Pandey A. (2020). Harnleiter und Hodenmetastase 25 Jahre nach Nephrektomie: Langjähriges Überleben mit einem metastasierten Nierenzellkarzinom [Ureteric and testicular mestastasis 25 years after nephrectomy: Long-term survival with metastatic renal cell carcinoma]. Der Urologe Ausg..

[30] Safriadi F., Noegroho B.S., Partogu B. (2020). Papillary renal cell carcinoma with testicular and penile metastases: A case report and literature review. Urol. Case Rep..

[31] Turco F., Tucci M., Di Stefano R.F., Samuelly A., Bungaro M., Bollito E., Scagliotti G.V., Buttigliero C. (2021). Are tyrosine kinase inhibitors an effective treatment in testicular metastases from kidney cancer? Case report. Tumori J..

[32] Moradi A., Farhoumand D., Bouzari B., Shakiba B. (2022). Simultaneous Bilateral Testicular Metastases from Renal Clear Cell Carcinoma: A Rare Presentation in Von Hippel–Lindau disease. J. Kidney Cancer VHL.

[33] Balawender K., Wawrzyniak A., Pliszka A., Rajda S., Walocha J., Wysiadecki G. (2023). Can renal cell carcinoma be encountered in male gonads? A rare primary manifestation of advanced kidney cancer?. Pol. Arch. Intern. Med..

[34] Thomson A., Park E.M., du Plessis J., Rachakonda K., Liodakis P. (2023). Rare presentation of a clear cell renal cell cancer metastasis to the contralateral testicle: A case report. Urol. Case Rep..

[35] Flanigan R.C., Campbell S.C., Clark J.I., Picken M.M. (2003). Metastatic renal cell carcinoma. Curr. Treat. Options Oncol..

[36] Choueiri T., Rini B., Garcia J., Baz R., Abou-Jawde R., Thakkar S., Elson P., Mekhail T., Zhou M., Bukowski R. (2007). Prognostic factors associated with long-term survival in previously untreated metastatic renal cell carcinoma. Ann. Oncol..

[37] Dudani S., de Velasco G., Wells C., Gan C.L., Donskov F., Porta C., Fraccon A., Pasini F., Hansen A.R., Bjarnason G.A. (2020). Sites of metastasis and survival in metastatic renal cell carcinoma (mRCC): Results from the International mRCC Database Consortium (IMDC). J. Clin. Oncol..

[38] Mikami S., Oya M., Mizuno R., Kosaka T., Katsube K.-I., Okada Y. (2014). Invasion and metastasis of renal cell carcinoma. Med. Mol. Morphol..

[39] Harding G., Cella D., Robinson D., Mahadevia P.J., Clark J., Revicki D.A. (2007). Symptom burden among patients with Renal Cell Carcinoma (RCC): Content for a symptom index. Health Qual. Life Outcomes.

[40] Ghazarian A.A., Rusner C., Trabert B., Braunlin M., McGlynn K.A., Stang A. (2018). Testicular cancer among US men aged 50 years and older. Cancer Epidemiol..

[41] Chen J., Daneshmand S. (2018). Modern Management of Testicular Cancer. Genitourinary Cancers.

[42] EAU Guidelines Office (2023). EAU Guidelines. Edn. Presented at the EAU Annual Congress Milan 2023.

[43] Gray R.E., Harris G.T. (2019). Carcinoma: Diagnosis and Management. Am. Fam. Physician.

